# Associations of urinary caffeine and caffeine metabolites with metabolic syndrome in US adults

**DOI:** 10.3389/fnut.2023.1280215

**Published:** 2023-12-01

**Authors:** Jianli Zhou, Linyuan Qin

**Affiliations:** ^1^Department of Science and Education, Guilin People’s Hospital, Guilin, China; ^2^Department of Epidemiology and Health Statistics, School of Public Health, Guilin Medical University, Guilin, China; ^3^Guangxi Key laboratory of Environmental Exposomics and Entire Lifecycle Health, Guilin, China

**Keywords:** caffeine, logistic regression, metabolic syndrome, principal components analysis, restricted cubic splines

## Abstract

**Aims:**

The relationship between caffeine and metabolic syndrome (MetS) has only been evaluated from the perspective of caffeine consumption. The association between urinary caffeine and MetS is still unclear. This study examined the associations between urinary caffeine and its metabolites and MetS and its components among adults.

**Methods:**

Data from the United States (US) National Health and Nutrition Examination Survey (NHANES) 2011–2014 was analyzed. NHANES is a stratified, multi-stage survey of all non-institutionalized persons in the US. A total of 2,394 subjects aged ≥ 18 years without missing data were selected in this study. Urinary caffeine and caffeine metabolite levels were quantified using high-performance liquid chromatography-electrospray ionization-tandem quadrupole mass spectrometry (HPLC-ESI-MS/MS) with stable isotope-labeled internal standards. We performed principal components analysis (PCA) to investigate the underlying correlation structure of 15 features of urinary caffeine and its metabolites and then used these principal components (PCs) as independent variables to conduct logistic regression analysis with or without restricted cubic spline (RCS) terms to explore the associations between caffeine metabolites and MetS.

**Results:**

Two main PCs that were derived from the PCA explained 90.67% of the total variance of caffeine and its metabolites. The first PC (PC1, strongly correlated with 1-MU, 1,3-DMU, 1,7-DMU, 1,3,7-TMU, 1-MX, 1,3-DMX, 1,7-DMX, 1,3,7-TMX, and AAMU) was positively correlated with risk of MetS (OR = 1.27, *p* < 0.001) and all its components (all ORs > 1, all *p*-values < 0.001) in the unadjusted models, while in the adjusted models, it was positively correlated with MetS (OR = 1.16, *p* = 0.042) and central obesity (OR = 1.22, *p* < 0.001). In the unadjusted model, there were significant associations between the second PC (PC2, correlated with 3-MU, 7-MU, 3,7-DMU, 3-MX, 7-MX, and 3,7-DMX) and MetS (OR = 1.11, *P* = 0.030) and central obesity (OR = 1.16, *P* < 0.001), while in the adjusted models (adjustment variables include gender, age, race/ethnicity, education level and income-poverty ratio, smoking status, drinking, and physical activity), PC2 was positively associated with MetS (OR = 1.15, *p* = 0.035) and central obesity (OR = 1.15, *p* = 0.005) and negatively associated with raised triglycerides (TG) (OR = 0.84, *p* = 0.008). Moreover, we observed U-shaped associations between PC1 and the risk of raised TG both in unadjusted (*P*_*non–linear*_ = 0.017) and adjusted (*P*_*non–linear*_ = 0.014) models.

**Conclusion:**

Urinary caffeine metabolites were positively associated with the risk of MetS and its components through different linear or non-linear patterns.

## 1 Introduction

Metabolic syndrome (MetS) is a multifactorial pathological condition with high prevalence among adult populations (1, 2); it includes central obesity, elevated triglycerides (TG), elevated blood pressure (BP), elevated fasting plasma glucose (FPG), and decreased high-density lipoprotein-cholesterol (HDL-C) (2–4). MetS is a major global public health problem and is associated with an increased risk of many diseases (5). Some studies have shown that MetS increases the risk of type 2 diabetes, cardiovascular disease, stroke, and myocardial infarction by two to five times (6, 7). Therefore, understanding the risk factors of MetS can decrease costs for public health systems and improve population health worldwide.

Coffee is one of the most popular drinks worldwide, with an estimated 165 million 60 kg bags consumed per year.^1^ Several studies have explored the relationship between coffee consumption and MetS, but the conclusions are inconsistent. For example, in 2016, a meta-analysis demonstrated that coffee consumption was associated with a low risk of MetS (8), and another study also demonstrated that coffee consumption was associated with a lower likelihood of having MetS (9). However, another meta-analysis including 297,817 individuals found no overall sex-adjusted association between coffee consumption and MetS (10). Besides, a non-linear relationship was found between coffee consumption and MetS in a previous study (8). Few studies have investigated caffeine, caffeine metabolites, and MetS. More than 70% of caffeine *in vivo* comes from coffee consumption, and 70–80% of caffeine is formed from 7-dimethylxanthine (84%), theobromine (12%), and theophylline (4%) through 3-N demethylation reaction catalyzed by CYP1A2 enzyme (11–13). Although no study has reported the relationships between caffeine and its metabolites and MetS, some studies have shown correlations between caffeine and caffeine metabolite with the risk of hypertension, insulin resistance, and cardiovascular disease (13–16), all of which may appear in people with MetS. A study reported that ambulatory systolic BP was inversely associated with urinary caffeine and its metabolites (13). Another study found that metabolites of caffeine, but not caffeine itself, significantly reduced the odds of hypertension in the US population (14). Additionally, a previous study indicated that levels of urinary caffeine, paraxanthine, and theophylline were associated with decreased parameters of arterial stiffness (17). A study conducted in the US showed that caffeine and caffeine metabolites were positively related to insulin resistance and beta cell function (15). Some researchers found that urinary theophylline and caffeine were inversely associated with cardiovascular diseases (CVDs) in women (16). Given the above inconsistent research and the non-linear relationship between coffee consumption and MetS reported by previous studies, we speculated that caffeine and its metabolites have linear or non-linear relationships with the risk of MetS and its components. In the present study, we sought to determine the associations of caffeine and its 14 caffeine metabolites with MetS and its components.

## 2 Materials and methods

### 2.1 Study population

The NHANES is a stratified, multi-stage survey of all non-institutionalized persons in the US. This study abstracted data from two consecutive cycles of the 2011–2014 NHANES examination. Individuals aged < 18 years and with missing data on one or more caffeine metabolites and MetS were excluded, resulting in a total of 2,394 subjects being included from both cycles. Missing data were not used for the analysis. The present study was exempt from formal ethics review as a secondary analysis of existing NHANES public data under the US Health and Human Services regulations at 45 CFR 46.101 (b). Ethical approval was given by the National Center for Health Statistics Ethics Review Board and all survey participants provided informed written consent.

### 2.2 Measurement of urinary caffeine and caffeine metabolite levels

Professional phlebotomists collected 24-h urine samples of participants. Specimens were stored under frozen (−20°C for short-term and −70°C for long-term storage) and light-avoided conditions until they were shipped to the National Center for Environmental Health for testing. The level of urine caffeine and 14 of its metabolites were quantified using high-performance liquid chromatography-electrospray ionization-tandem quadrupole mass spectrometry (HPLC-ESI-MS/MS) with stable isotope-labeled internal standards. The equipment used included the Agilent 1290 UHPLC system (Agilent Technologies, Palo Alta, CA, USA) and AB Sciex 6500 triple quad mass spectrometer (AB Sciex, Foster City, CA, USA). Positive and negative ionization modes were typically used for the analysis of caffeine and its metabolites. Kinetex 1.7 μ XB-C18 column 100 × 3.0 mm, 100 Å pore (Phenomenex, Torrance, CA, USA) was used for this analysis. The HPLC mobile phase system consisted of solvent A (aqueous): 5% methanol/0.05% formic acid and solvent B (organic)—90% methanol/0.05% formic acid. The gradient elution was employed to separate the compounds and the injection volume was 50 μL. More details of laboratory methodology are described in the caffeine and caffeine metabolites–urine lab procedure manual.^2^ Fifteen metabolites were measured, including 1-methyluric acid (1-MU), 3-methyluric acid (3-MU), 7-methyluric acid (7-MU), 1,3-dimethyluric acid (1,3-DMU), 1,7-dimethyluric acid (1,7-DMU), 3,7-dimethyluric acid (3,7-DMU), 1,3,7-trimethyluric acid (1,3,7-TMU), 1-methylxanthine (1-MX), 3-methylxanthine (3-MX), 7-methylxanthine (7-MX), theophylline (1,3-dimethylxanthine, 1,3-DMX), paraxanthine (1,7-dimethylxanthine, 1,7-DMX), theobromine (3,7-dimethylxanthine, 3,7-DMX), caffeine (1,3,7-trimethylxanthine, 1,3,7-TMX), and 5-acetylamino-6-amino-3-methyluracil (AAMU).

### 2.3 Definition of MetS

We defined MetS according to the new International Diabetes Federation^3^ as central obesity plus any two of the following four factors: (1) raised TG ≥ 150 mg/dL (1.7 mmol/L) or specific treatment for this lipid abnormality; (2) reduced HDL-C < 40 mg/dL (1.03 mmol/L) in men, < 50 mg/dL (1.29 mmol/L) in women, or specific treatment for this lipid abnormality; (3) raised BP: systolic BP ≥ 130 or diastolic BP ≥ 85 mm Hg or treatment of previously diagnosed hypertension; (4) raised FPG: ≥ 100 mg/dL (5.6 mmol/L) or previously diagnosed type 2 diabetes.

### 2.4 Covariates

Covariates were obtained from the NHANES 2011–2014 and included demographic characteristic factors (gender, age, race/ethnicity, education level, and income-poverty ratio) and health-related behavioral factors (smoking, drinking, physical activity, and body mass index [BMI]). Alcohol drinking over the past 12 months was used to assess alcohol consumption (CDC/NCHS, 2014). All these covariates have reached consensus in previous similar studies (16, 18). The details of the definition of covariates information were described in our previous study (19).

### 2.5 Statistical analysis

Levels of urinary caffeine and caffeine metabolites were Log10-transformed to correct distribution skewness. Median and quartiles were used to describe continuous data with a non-normal distribution, while frequency and percentage were used to describe categorical data. Spearman correlations were employed to test the relationship between urine caffeine and 14 caffeine metabolites. We used principal components analysis (PCA) as an exploratory analysis to investigate the underlying correlation structure of these 15 features. Orthogonal varimax rotation to the factors was applied, and we retained those with eigenvalues higher than one (20). PCA could transform highly correlated caffeine metabolites into a few unrelated principal components (PCs), such that the degrees of correlations between different PCs were low. Thus, the use of PCs as independent variables could reduce the multicollinearity among caffeine metabolites. PC scores (linear combinations of the caffeine and caffeine metabolites multiplied by their respective loadings) for each of the retained components were calculated for every individual. Then, we used these PCs as independent variables and MetS (and its components) as dependent variables for univariate logistic regression and multiple logistic regression analysis. Restricted cubic spline (RCS) logistic regression with three knots was used to explore the non-linear associations of the risk of MetS with levels of urinary caffeine and caffeine metabolites and the median values of levels of urinary caffeine and caffeine metabolites (21). Both the multiple logistic regression analysis and the RCS logistic regression were adjusted for the confounding demographic characteristic factors (gender, age, race/ethnicity, education level, and income-poverty ratio) and health-related behavioral factors (smoking, drinking, physical activity, and BMI). BMI was not considered as a confounding factor when the dependent variable was MetS or obesity. The “anova” function in the “rms” package (version 6.1-0) in R was used to estimate *P*_*overall*_ and *P*_*non–linear*_ to assess the statistical significance of dose-response associations. If both *p*-values were lower than 0.05, it indicated a non-linear dose-response association between the probability of MetS and the concentration of caffeine and caffeine metabolites in the urine. Due to the direct impact of renal function on the excretion of caffeine metabolites in urine, we conducted two sensitivity analyses to ascertain whether the renal function could influence the results. Specifically, we created two sub-datasets by excluding patients with an estimated glomerular filtration rate (eGFR) less than 60 mL/min per 1.73 m^2^ and an albumin-to-creatinine ratio greater than 30 mg/g, respectively. Subsequently, we performed logistic regression and RCS logistic regression models with these sub-datasets, with MetS and its components as the dependent variables [R Core Team (2023). R: A language and environment for statistical computing. R Foundation for Statistical Computing,^4^ Vienna, Austria.] and SPSS version 26 (SPSS Inc., Chicago, IL, USA) were used for all statistical analyses.

## 3 Results

### 3.1 Basic characteristics of the study population

The characteristics of this study population are presented in Table 1. Men represented 53.55% of the overall sample. Most of the participants were non-Hispanic white (39.60%). More than half (57.73%) of the participants had an educational level above 12 years. The median (P25∼P75) age of participants was 48.00 (33.00∼62.00) years. The median of non-log-transformed levels of urinary 1-MU, 3-MU, 7-MU, 1,3-DMU, 1,7-DMU, 3,7-DMU, 1,3,7-TMU, 1-MX, 3-MX, 7-MX, 1,3-DMX, 1,7-DMX, 3,7-DMX, 1,3,7-TMX, and AAMU were 56.85, 0.43, 11.10, 6.28, 24.40, 0.73, 1.24, 26.30, 22.95, 37.55, 1.65, 16.10, 13.45, 3.08, and 58.90 μmol/L, respectively.

**TABLE 1:** Characteristics of participants included in the study.

Characteristics	*N* (%) or median (P25 P75)
Categorical variables	*N* (%)
**Gender**	
Male	1,282 (53.55%)
Female	1,112 (46.45%)
**Race/ethnicity**	
Mexican American	273 (11.40%)
Other Hispanic	232 (09.69%)
Non-Hispanic white	948 (39.60%)
Non-Hispanic black	505 (21.09%)
Other	436 (18.21%)
**Education**	
< 12 years	517 (21.60%)
12 years	495 (20.68%)
> 12 years	1,382 (57.73%)
**Smoking status**	
Never	1,346 (56.22%)
Current	502 (20.97%)
Former	546 (22.81%)
**Physical activity**	
None	1,496 (62.49%)
Moderate	452 (18.88%)
Vigorous	446 (18.63%)
**Continuous variables**	**Median (P25 P75)**
Age, years	48.00 (33.00 62.00)
Income-poverty ratio	2.16 (1.07 4.16)
Drinking (times/year)	2.00 (1.00 4.00)
BMI	26.30 (23.10 30.10)
1-MU (umol/L)	56.85 (21.40 124.00)
3-MU (umol/L)	0.43 (0.14 1.17)
7-MU (umol/L)	11.10 (3.82 27.50)
1,3-DMU (umol/L)	6.28 (2.09 14.50)
1,7-DMU (umol/L)	24.40 (6.49 56.60)
3,7-DMU (umol/L)	0.73 (0.28 1.78)
1,3,7-TMU (umol/L)	1.24 (0.28 3.32)
1-MX (umol/L)	26.30 (9.13 64.00)
3-MX (umol/L)	22.95 (9.61 53.40)
7-MX (umol/L)	37.55 (15.50 87.90)
1,3-DMX (umol/L)	1.65 (0.53 3.45)
1,7-DMX (umol/L)	16.10 (4.83 33.60)
3,7-DMX (umol/L)	13.45 (5.46 30.40)
1,3,7-TMX (umol/L)	3.08 (0.77 8.24)
AAMU (umol/L)	58.90 (18.90 133.00)

1-MU, 1-methyluric acid; 3-MU, 3-methyluric acid; 7-MU, 7-methyluric acid; 1,3-DMU, 1,3-dimethyluric acid; 1,7-DMU, 1,7-dimethyluric acid; 3,7-DMU, 3,7-dimethyluric acid; 1,3,7-TMU, 1,3,7-trimethyluric acid; 1-MX, 1-methylxanthine; 3-MX, 3-methylxanthine; 7-MX, 7-methylxanthine; 1,3-DMX, 1,3-dimethylxanthine, theophylline; 1,7-DMX, 1,7-dimethylxanthine, paraxanthine; 3,7-DMX, 3,7-dimethylxanthine, theobromine; 1,3,7-TMX, 1,3,7-trimethylxanthine, caffeine; AAMU, 5-acetylamino-6-amino-3-methyluracil.

### 3.2 Principal component of caffeine and caffeine metabolites

**FIGURE 1:**
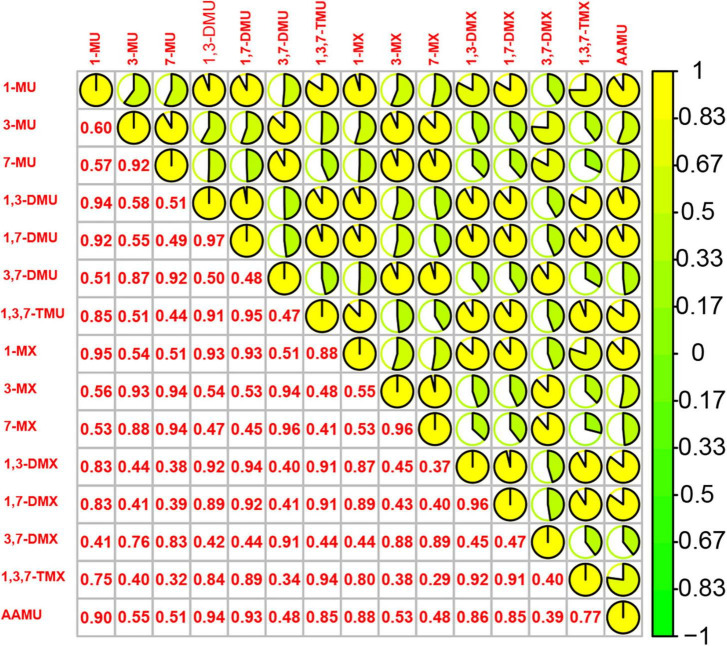
Spearman correlations between 15 urinary caffeine and caffeine metabolites. (1-MU: 1-methyluric acid; 3-MU: 3-methyluric acid; 7-MU: 7-methyluric acid; 1,3-DMU: 1,3-dimethyluric acid; 1,7-DMU: 1,7-dimethyluric acid; 3,7-DMU: 3,7-dimethyluric acid; 1,3,7-TMU: 1,3,7-trimethyluric acid; 1-MX: 1-methylxanthine; 3-MX: 3-methylxanthine; 7-MX: 7-methylxanthine, 1,3-DMX: 1,3-dimethylxanthine, theophylline; 1,7-DMX: 1,7-dimethylxanthine, paraxanthine; 3,7-DMX: 3,7-dimethylxanthine, theobromine; 1,3,7-TMX: 1,3,7-trimethylxanthine, caffeine; AAMU: 5-acetylamino-6-amino-3-methyluracil).

Spearman correlation analysis showed the significant relationships between urinary caffeine and caffeine metabolites; all coefficients (rs) were significant (Figure 1). The main PCs derived from the PCA are presented in Table 2. The first two components had eigenvalues ≥ 1 and accounted for 90.67% of the variance. Table 3 shows the rotated component matrix of urinary caffeine and 14 caffeine metabolites with correlations greater than 0.5. The first component (PC1) was strongly correlated with 1-MU, 1,3-DMU, 1,7-DMU, 1,3,7-TMU, 1-MX, 1,3-DMX, 1,7-DMX, 1,3,7-TMX, and AAMU. The second component (PC2) correlated with 3-MU, 7-MU, 3,7-DMU, 3-MX, 7-MX, and 3,7-DMX.

**TABLE 2:** Total variance showed by components of principal component analysis.

Component	Initial eigenvalues		
	Total	Proportion of variance (%)	Cumulative proportion (%)
1	11.07	73.77	73.77
2	2.54	16.91	90.67
3	0.59	3.92	94.59
4	0.23	1.56	96.14
5	0.14	0.9	97.04
6	0.1	0.65	97.69
7	0.09	0.59	98.27
8	0.08	0.52	98.79
9	0.05	0.36	99.15
10	0.04	0.28	99.43
11	0.03	0.17	99.6
12	0.02	0.14	99.74
13	0.02	0.11	99.85
14	0.02	0.1	99.95
15	0.01	0.05	100

**TABLE 3:** Rotated component matrix of 15 urinary caffeine and caffeine metabolites.

Caffeine metabolites	PC1	PC2
1-MU (umol/L)	0.830	
3-MU (umol/L)		0.845
7-MU (umol/L)		0.925
1,3-DMU (umol/L)	0.914	
1,7-DMU (umol/L)	0.922	
3,7-DMU (umol/L)		0.93
1,3,7-TMU (umol/L)	0.919	
1-MX (umol/L)	0.884	
3-MX (umol/L)		0.914
7-MX (umol/L)		0.942
1,3-DMX umol/L	0.933	
1,7-DMX umol/L	0.901	
3,7-DMX umol/L		0.857
1,3,7-TMX umol/L	0.910	
AAMU (umol/L)	0.872	

1-MU, 1-methyluric acid; 3-MU, 3-methyluric acid; 7-MU, 7-methyluric acid; 1,3-DMU, 1,3-dimethyluric acid; 1,7-DMU, 1,7-dimethyluric acid; 3,7-DMU, 3,7-dimethyluric acid; 1,3,7-TMU, 1,3,7-trimethyluric acid; 1-MX, 1-methylxanthine; 3-MX, 3-methylxanthine; 7-MX, 7-methylxanthine; 1,3-DMX, 1,3-dimethylxanthine, theophylline; 1,7-DMX, 1,7-dimethylxanthine, paraxanthine; 3,7-DMX, 3,7-dimethylxanthine, theobromine; 1,3,7-TMX, 1,3,7-trimethylxanthine, caffeine; AAMU, 5-acetylamino-6-amino-3-methyluracil.

### 3.3 Univariate logistic regression associations between MetS with PC scores of urinary caffeine metabolite levels

We used PC1 and PC2 as independent variables and investigated their associations with MetS and its components (Table 4). Effect sizes were different for the determinants across the two PCs. PC1 had significant positive associations with MetS and all components (all *p*-values < 0.001), and the models indicated that an increase in PC1 led to a 27, 25, 17, 23, 28, and 13% higher likelihood of MetS, raised FPG, raised BP, central obesity, raised TG, and reduced HDL-C, respectively. By contrast, PC2 had only significant positive associations with MetS and its components of central obesity (MetS: odds ratio [OR] = 1.11, 95% confidence interval [CI]: 1.01–1.22; central obesity: OR = 1.16, 95% CI: 1.08–1.25).

**TABLE 4:** Univariate logistic regression analysis of PC scores of Log10-transformed urinary caffeine and caffeine metabolites with MetS and its components.

Variables	OR (95% CI)	*p*-value
**MetS**		
PC 1	1.27 (1.15 ∼ 1.41)	**< 0.001**
PC2	1.11 (1.01 ∼ 1.22)	**0.03**
**Raised FPG**		
PC 1	1.25 (1.12 ∼ 1.39)	**< 0.001**
PC2	0.96 (0.87 ∼ 1.05)	0.352
**Raised BP**		
PC 1	1.17 (1.08 ∼ 1.26)	**< 0.001**
PC2	0.96 (0.89 ∼ 1.03)	0.268
**Central obesity**		
PC 1	1.23 (1.14 ∼ 1.33)	**< 0.001**
PC2	1.16 (1.08 ∼ 1.25)	**< 0.001**
**Raised TG**		
PC 1	1.28 (1.16 ∼ 1.42)	**< 0.001**
PC2	0.94 (0.86 ∼ 1.03)	0.192
**Reduced HDL-C**		
PC 1	1.13 (1.04 ∼ 1.21)	**0.002**
PC 2	1.03 (0.96 ∼ 1.11)	0.347

PC1: 1-MU; 1,3-DMU; 1,7-DMU; 1,3,7-TMU; 1-MX; 1,3-DMX; 1,7-DMX; 1,3,7-TMX; AAMU. PC2: 3-MU; 7-MU; 3,7-DMU; 3-MX; 7-MX; 3,7-DMX. Bold values indicates *P* < 0.05.

### 3.4 Multiple logistic regression associations between MetS with PC scores of levels of urinary caffeine metabolites levels

The effect sizes for the association of PC1 and PC2 with MetS and its components in the multiple logistic regression models are shown in Table 5. PC1 had a positive association with MetS (OR = 1.16, 95% CI: 1.01–1.34) and central obesity (OR = 1.22, 95% CI: 1.10–1.36). By contrast, PC2 had significant positive associations with MetS and central obesity but had a significant negative association with raised TG (all *p*-values < 0.001).

**TABLE 5:** Multiple logistic regression analysis of PC scores of log-transformed urinary caffeine and caffeine metabolites with MetS and its components.

Variables	OR (95% CI)	*P*-value
**MetS**		
PC1	1.16 (1.01 ∼ 1.34)	**0.042**
PC2	1.15 (1.01 ∼ 1.31)	**0.035**
**Raised FPG**		
PC1	1.15 (0.98 ∼ 1.34)	0.094
PC2	0.90 (0.78 ∼ 1.04)	0.155
**Raised BP**		
PC1	1.02 (0.90 ∼ 1.15)	0.795
PC2	0.94 (0.84 ∼ 1.05)	0.285
**Central obesity**		
PC1	1.22 (1.10 ∼ 1.36)	**< 0.001**
PC2	1.15 (1.04 ∼ 1.27)	**0.005**
**Raised TG**		
PC1	1.07 (0.92 ∼ 1.24)	0.375
PC2	0.84 (0.73 ∼ 0.95)	**0.008**
**Reduced HDL-C**		
PC1	0.93 (0.84 ∼ 1.03)	0.173
PC2	1.00 (0.91 ∼ 1.11)	0.949

PC1: 1-MU; 1,3-DMU; 1,7-DMU; 1,3,7-TMU; 1-MX; 1,3-DMX; 1,7-DMX; 1,3,7-TMX; AAMU. PC2: 3-MU; 7-MU; 3,7-DMU; 3-MX; 7-MX; 3,7-DMX. Bold values indicates *P* < 0.05.

### 3.5 Dose-response associations between MetS with PC scores of urinary caffeine metabolites levels

**FIGURE 2:**
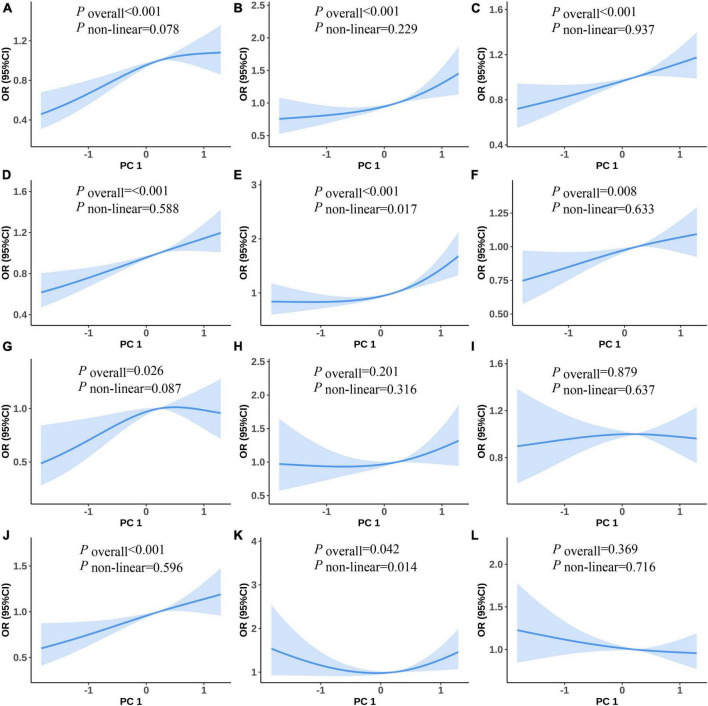
Predicted spline curves for the associations between the PC1 and MetS and its components using RCS logistic regression models [**(A–F)** and **(G–L)** show univariate and multiple RCS logistic regression analysis between PC1 and risk of MetS, raised FPG, raised BP, central obesity, raised TG, and reduced HDL-C, respectively].

Figure 2 shows the non-linear associations between the risk of PC1 and MetS and its components. Figures 2A–F show the RCS curves without adjustment for covariates and Figures 2G–J show the RCS curves with adjusting for covariates between PC1 and the risk of MetS, raised FPG, raised BP, central obesity, raised TG, and reduced HDL-C, respectively. We observed U-shaped associations between the risk of PC1 and raised TG in univariate and multiple RCS logistic regression models (Figures 2E, K, all values of *P*_*overall*_ and *P*_*non–linear*_ < 0.05). There were no statistically significant non-linear associations between PC1 and the risk of MetS and its other components in univariate and multiple RCS logistic regression models.

**FIGURE 3:**
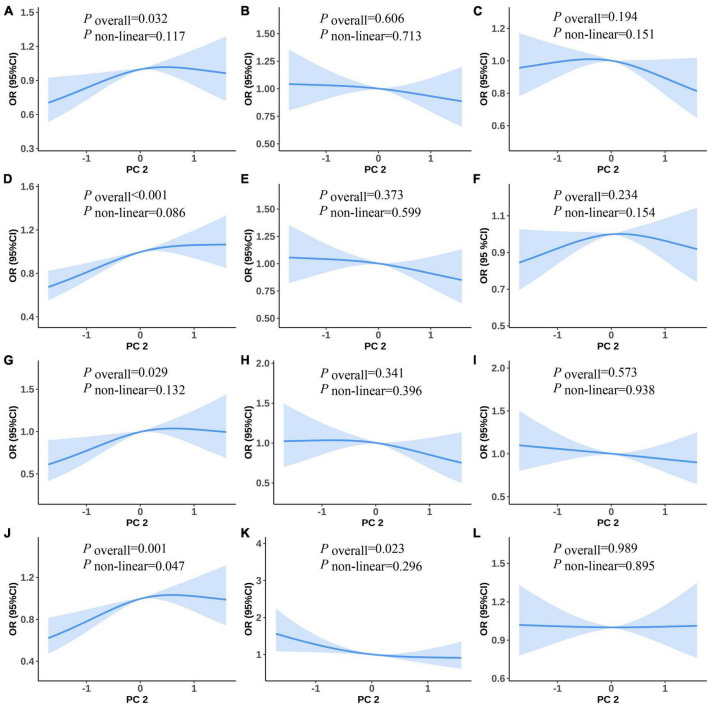
Predicted spline curves for the associations between the PC2 and MetS and its components using RCS logistic regression models [**(A–F)** and **(G–L)** show univariate and multiple RCS logistic regression analysis between PC2 and risk MetS, raised FPG, raised BP, central obesity, raised TG, and reduced HDL-C, respectively].

Figure 3 shows the non-linear associations between PC2 and the risk of MetS and its components. Figures 3A–F show the RCS curves without adjusting for covariates and Figures 3G–J show the RCS curves with adjusting for covariates for PC2 and the risk of MetS and its components. We did not observe any statistically significant non-linear associations between PC2 and the risk of MetS and its components in the univariate and multiple RCS logistic regression models (Figures 3A–L).

### 3.6 Sensitivity analysis

As evident from the adjusted results (Supplementary Tables 1, 2), the ORs of PC1 for MetS in both of the two sub-datasets and the full dataset were statistically significant with similar values, indicating a robust association between PC1 and MetS. However, for components of MetS, the OR of PC1 for central obesity was 1.22 (*p* < 0.001) in the full dataset, while in the sub-datasets, they were 1.14 (*p* = 0.067) and 1.12 (*p* = 0.116), respectively. The remaining MetS components exhibited robust results in both the full dataset and the sub-datasets. By contrast, the relationship between PC2 and MetS was not statistically significant in the two sub-datasets, but the ORs (1.14 and 1.10) were very close to the OR (1.15) obtained from the full dataset. This result indicated that the associations between PC2 and MetS components were robust.

In the adjusted RCS logistic regression analysis (Supplementary Figures 1, 2), the relationships between most of the dependent variables in the two sub-datasets and PC1 were consistent with the full dataset, indicating that the results were robust. However, in the sub-datasets, a non-linear relationship between central obesity and PC1 was observed, while the association was linear in the full dataset. Furthermore, in the sub-datasets, there was no association between raised TG and PC1 (*P*_*overall*_ and *P*_*non–linear*_ both > 0.05), which were consistent with the results of logistic regression. The relationships between PC2 and MetS and its components were mostly similar in the two sub-datasets (Supplementary Figures 3, 4). However, in the sub-datasets, PC2 showed a linear relationship with central obesity (*P*_*overall*_ < 0.05 and *P*_*non*–*linear*_ > 0.05) (Supplementary Figures 3J, 4J), whereas in the full dataset, the relationship was non-linear.

Overall, these findings suggested that the relationships between PC1 and MetS and its components remained robust in the sub-datasets, while the relationships between PC2 and MetS components showed some similarities but also slight differences compared to the full dataset.

## 4 Discussion

Caffeine is a xanthine alkaloid compound, which mainly exists in coffee, tea, cola, dark chocolate, and some analgesics (22, 23). Caffeine-containing diets are popular worldwide because of their effects in reducing fatigue, invigorating the spirit, and strengthening the stomach and the heart (24). Caffeine is a widely used psychoactive substance, often used as a stimulant of the central nervous system (25). However, in recent years, more and more research and epidemiological data have confirmed that caffeine is also important in energy metabolism (26, 27). In this study, we conducted a PCA of urinary caffeine and 14 caffeine metabolites and explored the linear and non-linear associations between PCs and the risk of MetS and its components among adults using data from NHANES 2011–2014. The PCA of 15 urinary caffeine and caffeine metabolites identified two main components that explained 90.67% of the total variance. Our study found that in the univariate logistic regression models, PC1 (strongly correlated with 1-MU, 1,3-DMU, 1,7-DMU, 1,3,7-TMU, 1-MX, 1,3-DMX, 1,7-DMX, 1,3,7-TMX, and AAMU) positively correlated with the risk of MetS and all its components, while in the multiple logistic regression models, it only positively correlated with the risk of MetS and central obesity. In the univariate logistic regression models, there were significant associations between PC2 (correlated with 3-MU, 7-MU, 3,7-DMU, 3-MX, 7-MX, and 3,7-DMX) and MetS and central obesity, while in the multiple logistic regression models, PC2 was positively correlated with the risk of MetS and central obesity but negatively correlated with raised TG. Moreover, we observed U-shaped associations between PC1 and raised TG in univariate and multiple logistic regression models. Our study further clarified the associations between caffeine exposure and the risk of MetS and its components, provided clues for the follow-up cohort study and mechanism study, and also provided a theoretical basis for the prevention and control of MetS.

The enzyme CYP1A2 in the human liver catalyzes the N-1, N-3, and N-7 demethylation of caffeine to form theobromine, paraxanthine, and theophylline, respectively (28). Our study showed that PC1 was highly associated with paraxanthine and theophylline pathways, and PC2 was highly associated with theobromine pathway (Supplementary Figure 5), indicating that these three pathways had different effects on MetS or its components.

Coffee and caffeine beverage consumption, which plays the most important role in caffeine intake, is influenced by socio-demographic factors such as age, gender, income, education, and smoking status (29, 30). To adjust for the confounding effects from these socio-demographic factors, we employed multiple logistic regressions to investigate the associations of caffeine and its metabolism in urine with MetS. To date, there has been little agreement on the associations between coffee consumption and MetS. It has previously been observed that in univariate and multiple logistic regression analyses, compared with non-coffee drinkers, Japanese men who drink ≥ 4 cups of coffee a day have a lower risk of developing MetS (31). This finding is consistent with another Japanese study that also found that coffee consumption was inversely correlated with the risk of MetS (18). These findings are contrary to another Korean study, which demonstrated that coffee consumption (particularly instant coffee mix) may have harmful effects on MetS (32). Our research showed that PC1 and PC2 were positively correlated with the risk of MetS in both univariate and multiple logistic models, which is consistent with the results of the Korean research. Even though no previous study has considered urinary caffeine and caffeine metabolites in relation to MetS, there are moderate correlations between coffee consumption and urinary caffeine (Spearman correlation = 0.437), paraxanthine (Spearman correlation = 0.528), and theophylline (Spearman correlation = 0.519), but there are no statistical correlations with other caffeine metabolites (33). Coffee is rich in phenolic acids, which have been shown to reduce the risk of MetS and alter the effects of caffeine on MetS (34). As a result, although our study is inconsistent with the effects of these studies, it is still comparable to them.

Metabolic syndrome (MetS) is a group of symptoms characterized by obesity, dyslipidemia, elevated BP, and impaired glucose regulation, and the relationships between caffeine (and caffeine metabolites) and MetS are affected by the components of MetS. Some studies have explored the relationships between caffeine and the risk of hypertension (13, 35, 36), but the results are still controversial. The study by Guessous (13) showed that inverse associations were observed for caffeine, 1,7-DMX, and 1,3-DMX with 24-h and night-time SBP, but no associations were observed between 3,7-DMX levels and 24-h or night-time ambulatory SBP. It has previously been observed that total plasma caffeine and 1,7-DMX at 10–13 weeks were inversely associated with glucose; this finding is contrary to our study that suggested that in the univariate model, PC1 was positively correlated with raised BP. Our study is consistent with the results of T R Hartley, which showed that caffeine raised both systolic and diastolic BP (35). We speculated that since caffeine is a methylxanthine and myocardial contractility can be enhanced by methylxanthines (36), this would increase cardiac output, thus raising BP. However, in the multiple logistic regression models, our study did not find statistical associations between caffeine and raised BP both in PC1 and PC2.

Data from the US adult study suggested that caffeine and its metabolites were positively related to insulin resistance (15). This is consistent with our results, which demonstrated that PC1 was positively associated with raised FPG in univariate logistic regression analysis. Previous studies have shown that caffeine consumption reduced insulin sensitivity in the short term (e.g., a 15% reduction after taking a dose of 3 mg per kilogram of body weight) (37). This may reflect a partly promoting effect of caffeine on the increased epinephrine release which may decrease the storage of glucose as glycogen in the muscle (38).

A prior study has shown that coffee consumption was not associated with the incidence of central obesity (39). In contrast to this finding, a Mendelian randomization study showed that high coffee consumption was associated with a low risk of obesity (40). A Korean study suggested that the elderly who consume less than one cup of coffee per day had a greater risk of sarcopenic obesity than those who consumed more than three cups per day (41). Up to now, far too little attention has been paid to the associations between caffeine and caffeine metabolites and central obesity. However, high levels of coffee consumption often indicate higher levels of caffeine and caffeine metabolites in the body, suggesting that these results are contrary to the results of our study. One explanation for these negative associations is that caffeine may improve energy balance by reducing appetite and increasing the basal metabolic rate and food-induced thermogenesis (42). Our study observed that there were positive associations between PC1/PC2 and central obesity in both univariate and multiple logistic regression models, indicating that the N-1, N-3, and N-7 demethylation of caffeine metabolic pathways were positively associated with obesity. As the components of PC1, methyluric acid, dimethyluric acid, and trimethyluric acid in caffeine metabolites have very similar molecular structures to uric acid, which causes mitochondrial oxidative stress that stimulates fat accumulation independent of excess calcium intake (43), we speculated that methyluric acid, dimethyluric acid, and trimethyluric acid may also have similar effects. Moreover, caffeine consumption is often accompanied by high energy intake, which may lead to excessive weight gain; this cofounder may affect the association between caffeine and obesity observed in our study.

Some epidemiological studies focused on the relationships between coffee and TG and HDL-C. A study conducted by Australian researchers suggested that coffee consumption was not associated with the risk of high TG or low HDL-C (39). Another study indicated no significant association between the consumption of coffee and serum lipid levels (44). However, a Japanese multi-institutional collaborative cohort study showed that coffee consumption was associated with lower serum TG levels (18). Besides, a Korean adult study demonstrated that in women, the prevalence of elevated TG and reduced HDL-C were significantly lower compared to non-coffee consumers (45). An ELSA-Brasil study indicated that more than three cups per day of coffee consumption was associated with a TG level increase (46). Our study found a negative association between PC2 (strongly correlated with 3-MU, 7-MU, 3,7-DMU, 3-MX, 7-MX, and 3,7-DMX) and raised TG in multiple logistic regression analysis, indicating the negative association of theobromine metabolic pathways with TG. Regarding PC1 (strongly correlated with 1-MU, 1,3-DMU, 1,7-DMU, 1,3,7-TMU, 1-MX, 1,3-DMX, 1,7-DMX, 1,3,7-TMX, and AAMU), we observed a non-linear positive relationship between PC1 and raised TG in the univariate and multiple logistic regression models although no linear relationship was observed between PC1 and raised TG. This indicates that the associations between caffeine metabolites and raised TG were not simple upward or downward relationships but complex non-linear relationships. In the previous randomized trial, high consumption of unfiltered coffee (median, six cups per day) increased low-density lipoprotein-cholesterol levels by 17.8 mg per deciliter compared with filtered coffee (47). Unfiltered coffee contains cafestol and kahweol, which increase the serum concentration of cholesterol and TG in humans (48); this may explain why caffeine and its metabolites were positively associated with raised TG.

To understand whether there was selection bias in our study, we compared the frequency distribution or median size of MetS, its components, and covariates between the caffeine metabolite missing population and the selected participants. As shown in Supplementary Table 3, the selected participants had lower prevalence rates of raised BP, central obesity, and raised TG. Except for alcohol consumption and smoking status, there were statistical differences in the other covariates between the two groups, and the selected participants had a higher income-poverty ratio and higher male frequency. There were statistical differences in other independent variables although the differences were moderate. This indicates that the findings of this study are more applicable to groups with a higher income-poverty ratio, a higher frequency of men, and a lower prevalence of raised BP, central obesity, and raised TG.

The main limitations of this study include its cross-sectional nature, preventing the elucidation of the causal relationship of urinary caffeine and its metabolites with MetS, and further prospective research is needed to verify the character of causation. Additionally, the participants in our study are all Americans, so the results of this study are not generally applicable to people in Asia or other parts of the world. However, the findings still have certain reference values because the NHANES cycle also includes some non-Hispanic and Asians.

Our study had some strengths. First, the biological components of different types of coffee could vary substantially, and urinary caffeine and caffeine metabolites are effective measures of caffeine consumption (11–13). Therefore, our study reflected the relationships between coffee and the risk of MetS more accurately compared with previous studies. Second, we used PCs as independent variables and MetS (and also its components) as dependent variables for the analysis; this, to some extent, could avoid the collinearity between caffeine and 14 kinds of caffeine metabolites. Third, besides exploring the linear associations between caffeine and its metabolites and the risk of MetS, our study further explored the non-linear relationships between caffeine and the risk of MetS.

To conclude, the elements of PC1 (1-MU, 1,3-DMU, 1,7-DMU, 1,3,7-TMU, 1-MX, 1,3-DMX, 1,7-DMX, 1,3,7-TMX, and AAMU) were positively correlated with MetS and central obesity. The elements of PC2 (3-MU, 7-MU, 3,7-DMU, 3-MX, 7-MX, and 3,7-DMX) were positively correlated with the risk of MetS and central obesity and negatively correlated with raised TG. There were U-shaped associations between PC1 and the risk of elevated TG in univariate and multiple RCS logistic regression models. Further prospective studies are required to confirm the causal associations between caffeine metabolites and MetS. Our study provides empirical support for formulating an acceptable concentration range of caffeine metabolites in urine in the future and offers a new concept for preventing the occurrence of MetS.

## Data availability statement

Publicly available datasets were analyzed in this study. This data can be found here: https://www.cdc.gov/nchs/nhanes/index.htm.

## Ethics statement

The present study was exempt from formal ethics review as a secondary analysis of existing NHANES public data under the US Health and Human Services (HHS) regulations at 45 CFR 46.101 (b). The NHANES surveys were approved by the US National Center for Healthcare Statistics (NCHS) Research Ethics Review Board (ERB), and the NCHS IRB/ERB protocol number for NHANES 2011–2012 was protocol #2011-17 and NHANES 2013–2014 was continuation of protocol #2011-17. Participants gave written informed consent before the home interview and health exams. The studies were conducted in accordance with the local legislation and institutional requirements. The participants provided their written informed consent to participate in this study.

## Author contributions

JZ: Conceptualization, Data curation, Investigation, Methodology, Validation, Visualization, Writing – original draft, Writing – review & editing. LQ: Conceptualization, Data curation, Investigation, Methodology, Validation, Visualization, Writing – review & editing.

## Footnotes

1
https://www.statista.com/statistics/292595/global-coffee-consumption/

2
https://wwwn.cdc.gov/nchs/data/nhanes/2013-2014/labmethods/CAFE-H-MET-508.pdf

3
https://idf.org/about-diabetes/resources/?type=364

4
http://www.R-project.org/
