# Supplementary material for: Associations of urinary caffeine and caffeine metabolites with metabolic syndrome in US adults
Source: Front Nutr. 2023 Dec 1;10:1280215. doi: 10.3389/fnut.2023.1280215 (PMC10722267; doi:10.3389/fnut.2023.1280215)
Supplement: Supplementary file 1 [file Data_Sheet_1.docx]

Electronic Supporting Materials

**Associations of urinary caffeine and caffeine metabolites**

**with metabolic syndrome in U.S. adults**

Jianli Zhou ^a, 1*^, Linyuan Qin ^b, c,1^,

^a^ Department of Science and Education, Guilin People’s Hospital, Guilin, 541000, P. R. China.

^b^ Department of Epidemiology and Health Statistics, School of Public Health, Guilin Medical University, Guilin, 541000, P. R. China.

^c^ Guangxi key laboratory of Environmental Exposomics and Entire Lifecycle Health, Guilin, 541000, P. R. China.

*Corresponding author: Jianli Zhou, Department of Science and Education, Guilin People’s Hospital, No. 12 Wenming Road, Guilin, Guangxi, China, Tel: +86-0773-2882100; fax: +86-0773-2881579. Email: [jianliz555@163.com](mailto:jianliz555@163.com).

^1^ Jianli Zhou, Linyuan Qin contributed equally to this work

**Supporting Tables**

**Table** **S1** Univariate logistic regression analysis of PC scores of Log10-transformed urinary caffeine and caffeine metabolites with MetS and its components exclude participants with eGFR less than 60 ml/min per 1.73 m^2^ or ACR greater than 30 mg/g.

| Variables | Participants without eGFR less than 60 ml/min per 1.73 m^2^ (n= 2219) | |  | Participants without ACR greater than 30 mg/g (n= 2106) | |
| --- | --- | --- | --- | --- | --- |
|  | OR (95% CI) | *P* value |  | OR (95% CI) | *P* value |
| **MetS** |  |  |  |  |  |
| PC 1 | 1.29 (1.15~1.45) | **<0.001** |  | 1.27 (1.13~1.43) | **<0.001** |
| PC 2 | 1.11 (1.01~1.23) | **0.038** |  | 1.11 (1.00~1.24) | **0.046** |
| **Raised FPG** |  |  |  |  |  |
| PC 1 | 1.25 (1.11~1.41) | **<0.001** |  | 1.20 (1.06~1.36) | **0.005** |
| PC 2 | 0.95 (0.85~1.06) | 0.374 |  | 0.95 (0.85~1.06) | 0.353 |
| **Raised BP** |  |  |  |  |  |
| PC 1 | 1.22 (1.10~1.34) | **<0.001** |  | 1.20 (1.08~1.32) | **0.001** |
| PC 2 | 0.97 (0.88~1.06) | 0.490 |  | 1.00 (0.91~1.10) | 0.982 |
| **Central obesity** |  |  |  |  |  |
| PC 1 | 1.18 (1.07~1.30) | **0.001** |  | 1.16 (1.05~1.29) | **0.004** |
| PC 2 | 1.17 (1.06~1.28) | **0.001** |  | 1.15 (1.05~1.27) | **0.003** |
| **Raised TG** |  |  |  |  |  |
| PC 1 | 1.37 (1.21~1.55) | **<0.001** |  | 1.30 (1.15~1.47) | **<0.001** |
| PC 2 | 0.89 (0.80~0.99) | **0.032** |  | 0.91 (0.82~1.02) | 0.097 |
| **Reduced HDL-C** |  |  |  |  |  |
| PC 1 | 1.15 (1.04~1.27) | **0.005** |  | 1.12 (1.01~1.24) | **0.028** |
| PC 2 | 0.97 (0.88~1.06) | 0.496 |  | 0.96 (0.87~1.05) | 0.365 |

**Table S2** Multiple logistic regression analysis of PC scores of Log10-transformed urinary caffeine and caffeine metabolites with MetS and its components exclude participants with eGFR less than 60 ml/min per 1.73 m^2^ or ACR greater than 30 mg/g.

| Variables | Participants without eGFR less than 60 ml/min per 1.73 m^2^ (n= 2219) | |  | Participants without ACR greater than 30 mg/g (n= 2106) | |
| --- | --- | --- | --- | --- | --- |
|  | OR (95% CI) | *P* value |  |  | *P* value |
| **MetS** |  |  |  |  |  |
| PC 1 | 1.21 (1.03~1.41) | **0.017** |  | 1.18 (1.01~1.39) | **0.042** |
| PC 2 | 1.14 (0.99~1.31) | 0.076 |  | 1.10 (0.96~1.27) | 0.182 |
| **Raised FPG** |  |  |  |  |  |
| PC 1 | 1.11 (0.93~1.32) | 0.243 |  | 1.06 (0.89~1.26) | 0.547 |
| PC 2 | 0.92 (0.78~1.07) | 0.264 |  | 0.86 (0.74~1.01) | 0.066 |
| **Raised BP** |  |  |  |  |  |
| PC 1 | 1.04 (0.90~1.21) | 0.600 |  | 1.04 (0.89~1.21) | 0.620 |
| PC 2 | 1.01 (0.88~1.17) | 0.840 |  | 1.01 (0.88~1.16) | 0.931 |
| **Central obesity** |  |  |  |  |  |
| PC 1 | 1.14 (0.99~1.30) | 0.067 |  | 1.12 (0.97~1.29) | 0.116 |
| PC 2 | 1.15 (1.02~1.31) | **0.025** |  | 1.14 (1.00~1.29) | **0.048** |
| **Raised TG** |  |  |  |  |  |
| PC 1 | 1.14 (0.96~1.35) | 0.144 |  | 1.09 (0.92~1.30) | 0.331 |
| PC 2 | 0.79 (0.68~0.92) | **0.003** |  | 0.77 (0.66~0.90) | **0.001** |
| **Reduced HDL-C** |  |  |  |  |  |
| PC 1 | 0.96 (0.84~1.10) | 0.531 |  | 0.94 (0.82~1.08) | 0.402 |
| PC 2 | 0.93 (0.82~1.05) | 0.253 |  | 0.90 (0.79~1.02) | 0.096 |

**Table S3** Characteristics of participants included in this study and participants with missing data on one or more caffeine metabolites.

| Characteristics | Eligible participants included in this study | Participants with missing data on one or more caffeine metabolites |  |  |
| --- | --- | --- | --- | --- |
| **Categorical variables** | **N (%)** | **N (%)** | **χ^2^** | ***P* value** |
| **Gender** |  |  |  |  |
| Male | 1282 (53.55%) | 4040 (48.12%) | 21.95 | <0.001 |
| Female | 1112 (46.45%) | 4355 (51.88%) |  |  |
| **Race/ethnicity** |  |  |  |  |
| Mexican American | 273 (11.40%) | 1014 (12.08%) | 12.62 | 0.013 |
| Other Hispanic | 232 (09.69%) | 803 (09.57%) |  |  |
| Non-Hispanic white | 948 (39.60%) | 3274 (39.00%) |  |  |
| Non-Hispanic black | 505 (21.09%) | 1979 (23.57%) |  |  |
| Other | 436 (18.21%) | 1325 (15.78%) |  |  |
| **Education** |  |  |  |  |
| <12 y | 517 (21.60%) | 1860 (23.51%) | 7.34 | 0.025 |
| 12 y | 495 (20.68%) | 1730 (21.87%) |  |  |
| >12 y | 1382 (57.73%) | 4321 (54.62%) |  |  |
| **Smoking status** |  |  |  |  |
| Never | 1346 (56.22%) | 4708 (57.63%) | 1.72 | 0.423 |
| Current | 502 (20.97%) | 1629 (19.94%) |  |  |
| Former | 546 (22.81%) | 1833 (22.44%) |  |  |
| **Physical activity** |  |  |  |  |
| None | 1496 (62.49%) | 5397 (64.29%) | 9.48 | 0.009 |
| Moderate | 452 (18.88%) | 1656 (19.73%) |  |  |
| Vigorous | 446 (18.63%) | 1342 (15.99%) |  |  |
| **MetS** |  |  |  |  |
| no | 1713 (71.55%) | 3960 (72.28%) | 0.43 | 0.511 |
| yes | 681 (28.45%) | 1519 (27.72%) |  |  |
| **Raised FPG** |  |  |  |  |
| no | 737 (44.56%) | 1874 (43.77%) | 0.3 | 0.585 |
| yes | 917 (55.44%) | 2407 (56.23%) |  |  |
| **Raised BP** |  |  |  |  |
| no | 1234 (53.03%) | 3740 (49.50%) | 8.85 | 0.003 |
| yes | 1093 (46.97%) | 3815 (50.50%) |  |  |
| **Central obesity** |  |  |  |  |
| no | 1444 (60.55%) | 3334 (44.04%) | 197.89 | <0.001 |
| yes | 941 (39.45%) | 4236 (55.96%) |  |  |
| **Raised TG** |  |  |  |  |
| no | 879 (51.22%) | 2181 (46.10%) | 13.26 | <0.001 |
| yes | 837 (48.78%) | 2550 (53.90%) |  |  |
| **Reduced HDL-C** |  |  |  |  |
| no | 1383 (57.77%) | 4723 (56.26%) | 1.73 | 0.189 |
| yes | 1011 (42.23%) | 3672 (43.74%) |  |  |
| **Continuous variables** | **Median [IQR]** | **Median [IQR]** | **Z** | ***P* value** |
| **Age** (years) | 48.00 [33.00~62.00] | 47.00 [31.00~63.00] | -2.07 | 0.038 |
| **Income-poverty ratio** | 2.16 [1.07~4.16] | 1.91 [0.97~3.94] | -3.51 | <0.001 |
| **Drinking** (times/year) | 2.00 [1.00~4.00] | 2.00 [1.00~4.00] | -0.87 | 0.382 |

**Supporting Figures**


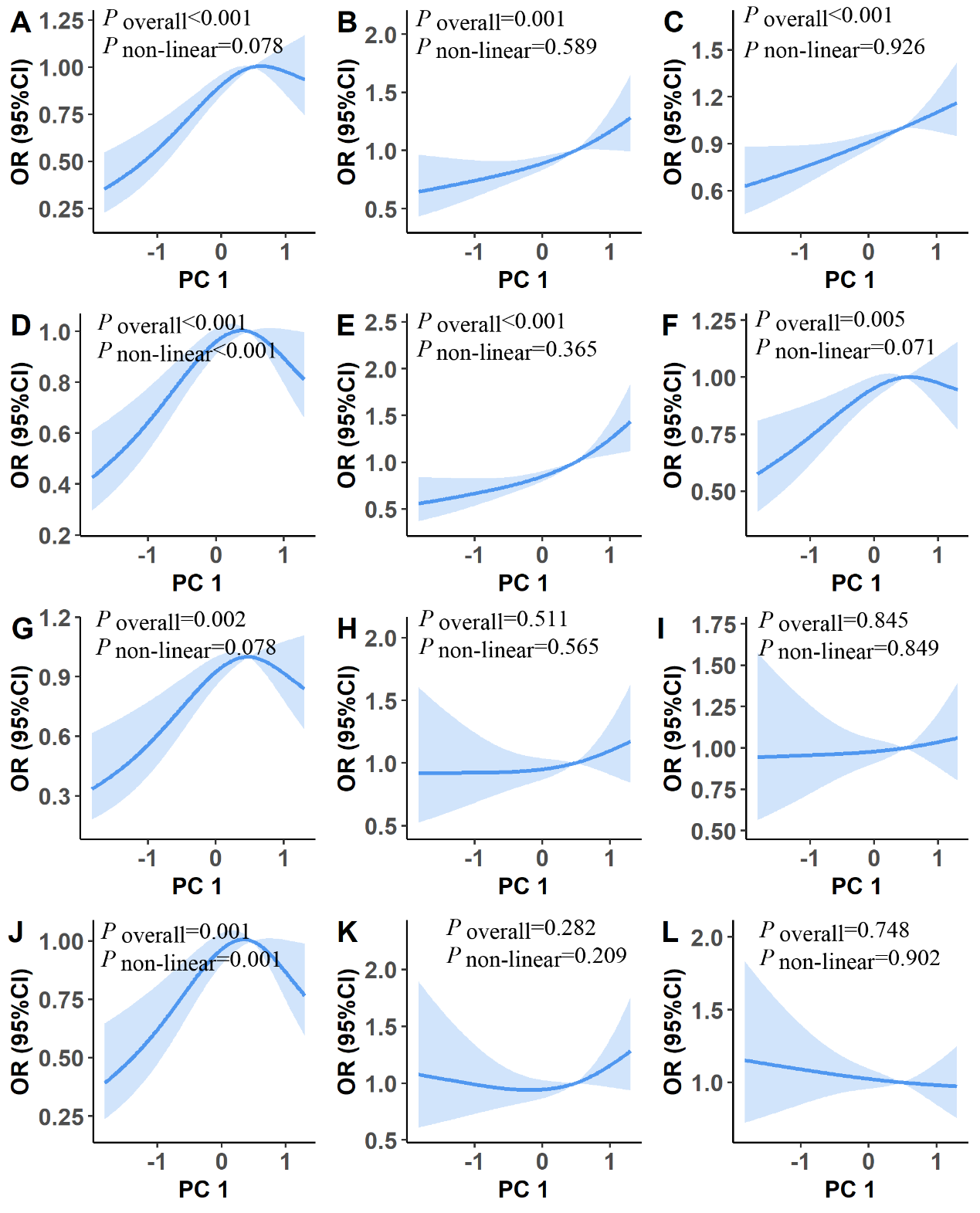


# Figure S1 Predicted spline curves for the associations between the PC1 and MetS and its components using RCS logistic regression models (participants without eGFR less than 60 ml/min per 1.73 m^2^). (Figure S1 A~ F and G ~ L show univariate and multiple RCS logistic regression analysis between PC1 and risk of MetS, raised FPG, raised BP, central obesity, raised TG and reduced HDL-C, respectively).


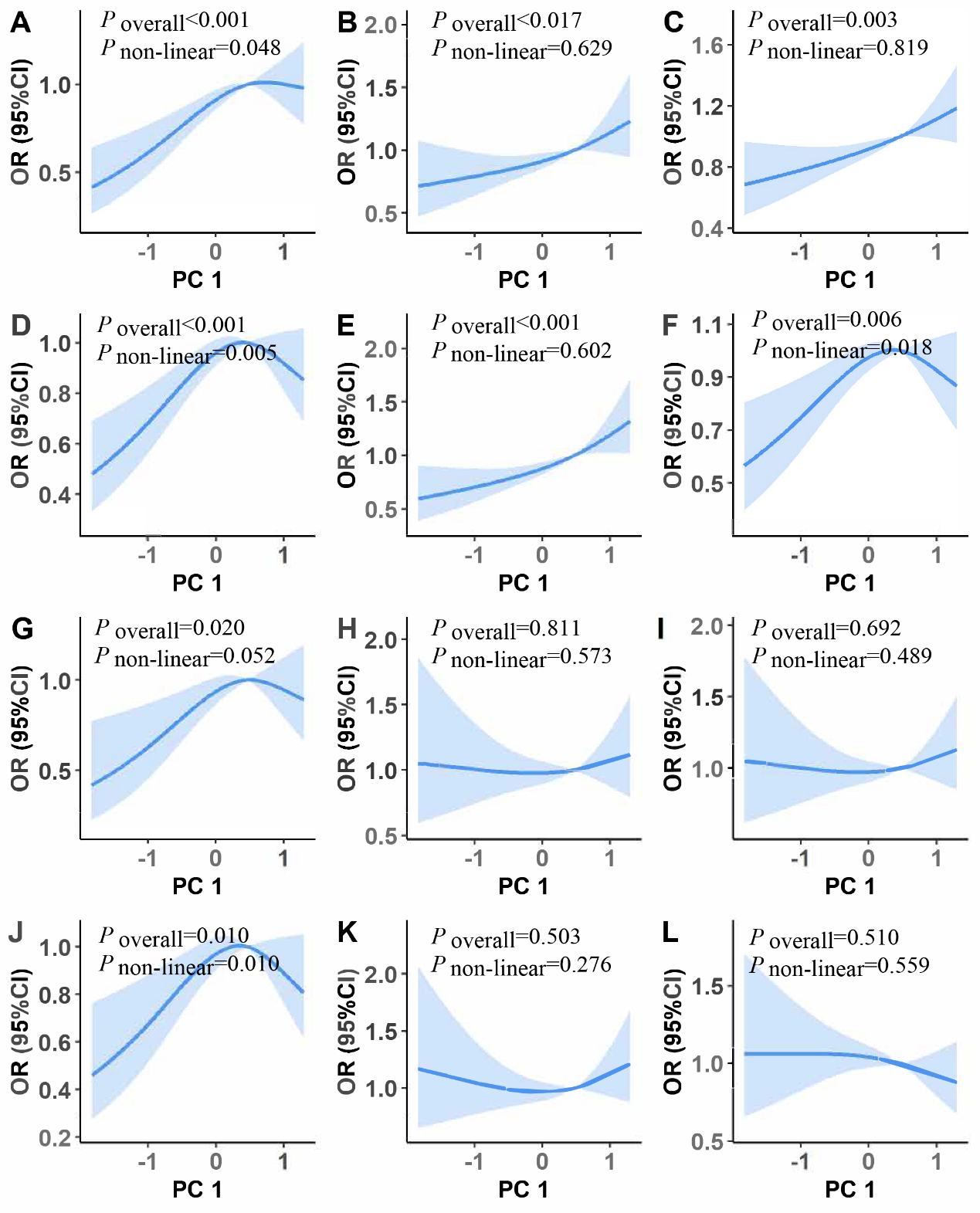


**Figure S2** Predicted spline curves for the associations between the PC1 and MetS and its components using RCS regression models (participants without ACR greater than 30 mg/g**)**. **(**Figure S2 A~ F and G ~ L show univariate and multiple RCS logistic regression analysis between PC1 and risk of MetS, raised FPG, raised BP, central obesity, raised TG and reduced HDL-C, respectively**).**


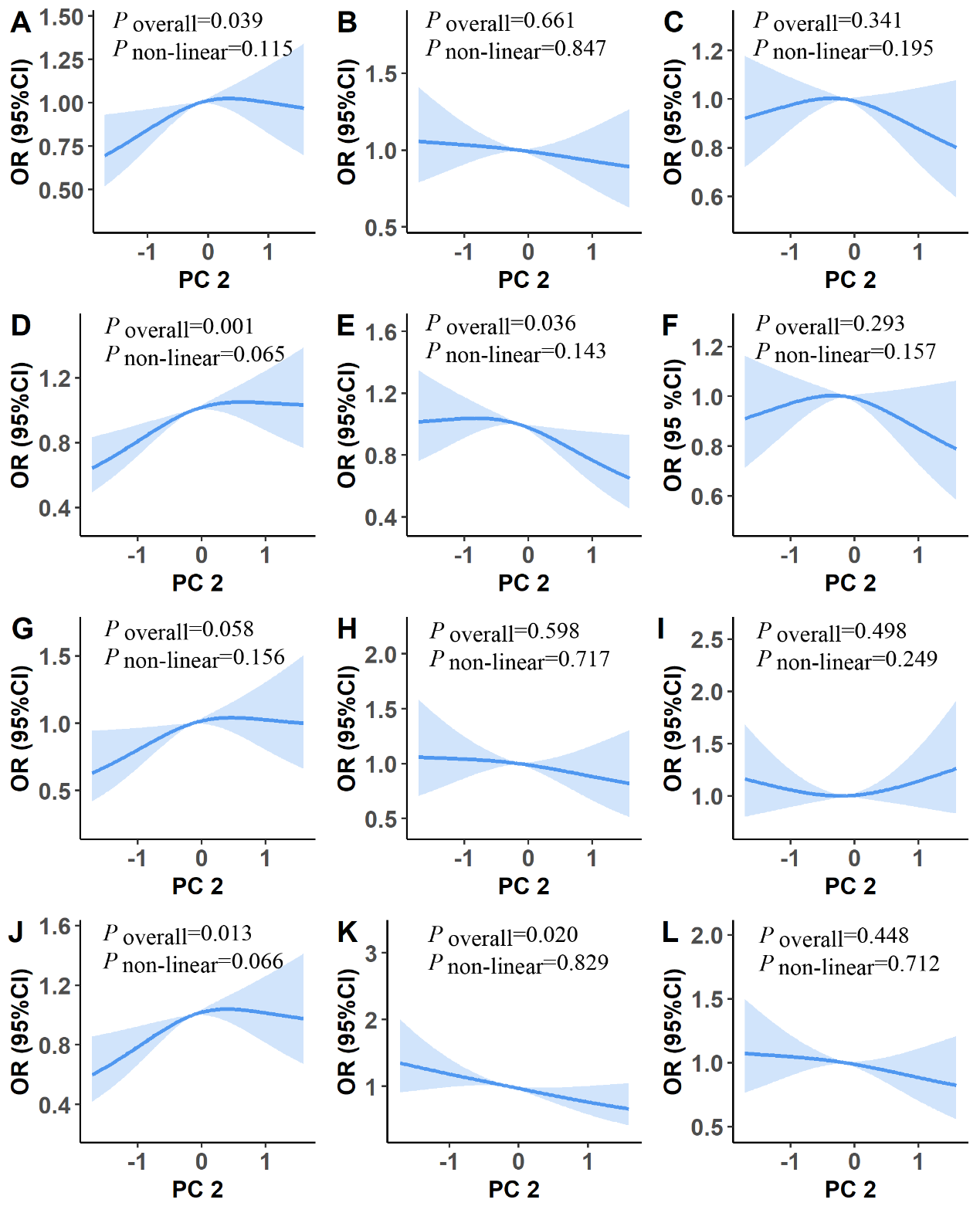


# Figure S3 Predicted spline curves for the associations between the PC2 and MetS and its components using RCS regression models (participants without eGFR less than 60 ml/min per 1.73 m^2^). (Figure S3 A~ F and G ~ L show univariate and multiple RCS logistic regression analysis between PC2 and risk of MetS, raised FPG, raised BP, central obesity, raised TG and reduced HDL-C, respectively).


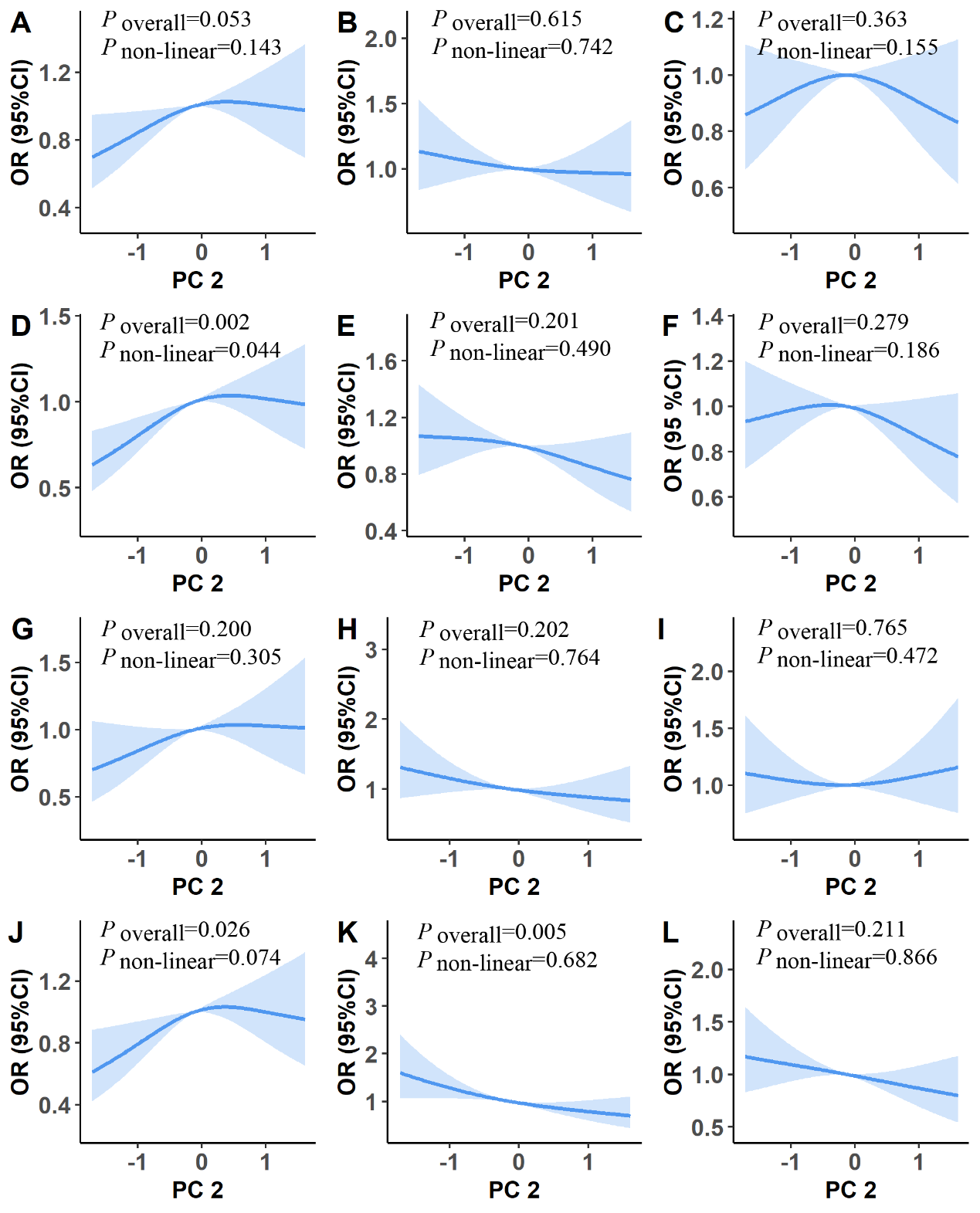


**Figure S4** Predicted spline curves for the associations between the PC2 and MetS and its components using RCS logistic regression models (participants without ACR greater than 30 mg/g**)**. **(**Figure S4 A~ F and G ~ L show univariate and multiple RCS logistic regression analysis between PC2 and risk of MetS, raised FPG, raised BP, central obesity, raised TG and reduced HDL-C, respectively**).**


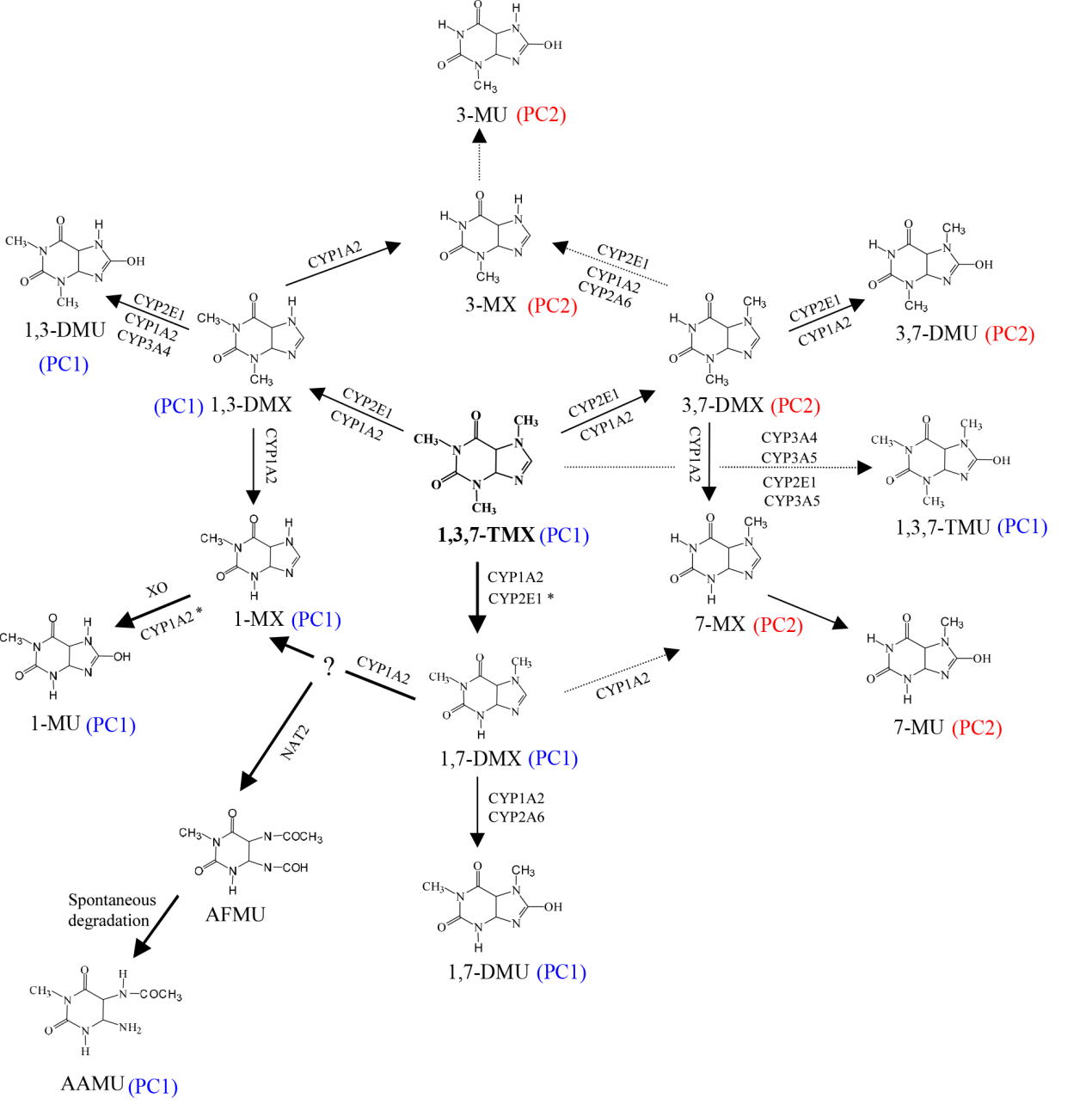


**Figure S5** Caffeine metabolism pathways in human liver (1). Bold arrows point out main pathways and dotted arrows mark minor pathways, which do not contribute to any of the ratios used to mark CYP1A2 or NAT2 activity. Symbols beside arrows indicate enzymes: NAT2, N-acetyltransferase; XO, xanthine oxidase. 1-MU: 1-methyluric acid; 3-MU: 3-methyluric acid; 7-MU: 7-methyluric acid; 1,3-DMU: 1,3-dimethyluric acid; 1,7-DMU: 1,7-dimethyluric acid; 3,7-DMU: 3,7-dimethyluric acid; 1,3,7-TMU: 1,3,7-trimethyluric acid; 1-MX: 1-methylxanthine; 3-MX: 3-methylxanthine; 7-MX: 7-methylxanthine, 1,3-DMX: 1,3-dimethylxanthine, theophylline; 1,7-DMX: 1,7-dimethylxanthine, paraxanthine; 3,7-DMX: 3,7-dimethylxanthine, theobromine; 1,3,7-TMX: 1,3,7-trimethylxanthine, caffeine; AAMU: 5-acetylamino-6-amino-3-methyluracil.

**References**

1. Caubet MS, Elbast W, Dubuc MC, Brazier JL. Analysis of urinary caffeine metabolites by HPLC-DAD: the use of metabolic ratios to assess CYP1A2 enzyme activity. J Pharm Biomed Anal. (2002) 27(1-2):261-70. doi:10.1016/s0731-7085(01)00546-5.
